# Generalizable preconditioning strategies for MAP PET reconstruction using Poisson likelihood

**DOI:** 10.3389/fnume.2025.1661332

**Published:** 2025-12-02

**Authors:** Matteo N. Colombo, Marco Paganoni, Luca Presotto

**Affiliations:** Department of Physics “Giuseppe Occhialini”, University of Milano-Bicocca, Milano, Italy

**Keywords:** image reconstruction, tomography, preconditioning, Poisson likelihood, optimization, positron emission tomography

## Abstract

**Introduction:**

The positron emission tomography (PET) problem with Poisson log-likelihood is notoriously ill-conditioned. This stems from its dependence on the inverse of the measured counts and the square of the attenuation factors, causing the diagonal of the Hessian to span over 5 orders of magnitude. Optimization is, therefore, slow, motivating decades of research into acceleration techniques. In this paper, we propose a novel preconditioner tailored for maximum *a posteriori* (MAP) PET reconstruction priors that is designed to achieve approximately uniform spatial resolution.

**Methods:**

Our approach decomposes the Hessian into two components: one diagonal and one circulant. The diagonal term is the Hessian expectation computed in an initial solution estimate. As the circulant term, we use an apodized 2D ramp filter. We evaluated our method on the PET Rapid Image reconstruction Challenge dataset that includes a wide range of phantoms, scanner models, and count levels. We also varied the regularization strengths. Our preconditioner was implemented in a conjugate gradient descent algorithm without subsets or stochastic acceleration.

**Results:**

We show that the proposed preconditioner consistently achieves convergence in fewer than 10 full iterations—each consisting of just one forward and one backward projection. We also show that the circulant component, despite its crude 2D approximation, provides very meaningful acceleration beyond the diagonal-only case.

**Discussion:**

These results demonstrate that decomposing the Hessian into diagonal and circulant components is an effective strategy for accelerating MAP PET reconstruction. The proposed preconditioner significantly improves convergence speed in challenging, ill-conditioned Poisson PET inverse problems.

## 1 Introduction

Positron emission tomography (PET) plays a crucial role in modern medical imaging, offering functional insights into tissue metabolism and receptor activity. However, accurate image reconstruction from PET data remains a mathematically ill-posed and computationally demanding inverse problem (1). Indeed, the favored approach is the Bayesian framework; with the acquisition modeled as a Poisson process and the possibility to include *a priori* information. However, the curvature of the Poisson log-likelihood makes the optimization especially difficult (2, 3). In this work, we investigate a novel preconditioning approach to accelerate maximum *a posteriori* (MAP) image reconstruction, which, being generalizable, can be combined with further acceleration techniques.

### 1.1 The PET reconstruction problem

In emission tomography, individual events are recorded in each detector bin; therefore, the data follow the Poisson statistics. In positron emission tomography, detector bins are represented by lines of response (LORs), each characterized by an attenuation coefficient, $a_{i}$, that represents the probability of both photons reaching the detector without being attenuated. In PET, this coefficient is independent of the emission position along the LOR. In this article, we will refer to the log-likelihood of the data as $-\log p(y|\lambda )=-L(\lambda )=\sum_{i}\bar{y_{i}}-y_{i}\ln\bar{y_{i}}$, where $y_{i}$ is the number of events recorded in LOR i, while $\bar{y_{i}}$ is the number of expected counts in the same LOR, given an image λ; the two are related by $\bar{y_{i}}=\sum_{j}a_{i}\;c_{ij}\;\lambda_{j}+b_{i}$, where $a_{i}$ is the aforementioned attenuation factor, $c_{ij}$ is the probability that a positron emission starting in pixel j and not attenuated in the body is detected as a coincidence in LOR i, and $b_{i}$ is the number of expected background counts in that bin (i.e., the sum of the random and scatter corrections). Note that it is possible to include the factor $a_{i}$ in $c_{ij}$.

In PET, penalized maximum likelihood image reconstruction algorithms have emerged as a powerful method class (4, 5). Their main advantage lies in the possibility of incorporating accurate statistical models of the acquisition process together with prior information, which makes it possible to simultaneously encourage the preservation of smooth regions while still promoting sharp edges and clear structural boundaries. This balance often translates into images with improved diagnostic quality, reduced noise amplification, and enhanced robustness with respect to measurement variability. In the Bayesian framework, these algorithms are interpreted as MAP estimation, where the goal is to reconstruct λ by maximizing the posterior distribution log⁡p(λ|y)=log⁡p(y|λ)+log⁡p(λ). The prior p(λ) introduces a regularization, typically through a penalty term R(λ). In practice, MAP estimation leads to the minimization of a penalized objective function, as shown in the following equation:

$$\Phi (\lambda )=-L(\lambda )+\beta R(\lambda );\;\lambda_{MAP}=\arg\min_{\lambda \geq 0}\left[\Phi (\lambda )\right],$$ (1)

The parameter β in Equation 1 controls the tradeoff between resolution and noise.

The gradient and Hessian of the tomographic log-likelihood are given by the following equations, respectively:

$$\frac{\partial L}{\partial \lambda_{j}}=\sum_{i}a_{i}\;c_{ij}\left(\frac{{\bar{y}}_{i}-y_{i}}{{\bar{y}}_{i}}\right),\;\frac{\partial^{2}L}{\partial \lambda_{j}\partial \lambda_{k}}=\sum_{i}a_{i}^{2}\;\frac{y_{i}}{{\bar{y_{i}}}^{2}}\;c_{ij}\;c_{ik}.$$ (2)

Equation 2 highlights immediately two issues in the Hessian. As $a_{i}\in [∼0.01,1]$ in a field of view containing a human adult abdomen, the presence of this value at the second power already shows how the diagonal of the Hessian typically spans more than 4 orders of magnitude. Furthermore, even the expectation of $\left(y_{i}/{\bar{y_{i}}}^{2}\right)$ $E\left(y_{i}/{\bar{y_{i}}}^{2}\right)=1/\bar{y_{i}}$ spans 1 or more orders of magnitude when moving from LORs that intersect the majority of the body to purely background LORs. All of these factors make the convergence of algorithms maximizing the gradient of the Poisson likelihood especially slow.

For MAP PET reconstruction, the state-of-the-art approaches include gradient-based methods, primal-dual algorithms, and surrogate techniques. To accelerate computation, subset strategies, such as ordered subset expectation maximization (OSEM) (6) and block sequential regularized expectation maximization (BSREM) (7), are widely used, though they may suffer from convergence issues. More recently, stochastic variance reduction (SVR) methods, including stochastic variance reduced gradient (SVRG), stochastic average gradient (SAG), and SAGA (8), have been introduced to reduce gradient variance and allow more stable convergence toward the MAP solution (9). These algorithms work by storing and utilizing previously computed subset gradients to improve the accuracy of the update direction. The SVR techniques have been shown to reduce variations in successive image updates and can achieve faster convergence to the penalized maximum likelihood solution under suitable conditions, while still relying on gradient-based updates.

All the previously listed techniques mainly focus on stochastic-based acceleration. Another independent strategy to achieve fast convergence is preconditioning. This modifies the optimization problem so that the effective Hessian becomes better conditioned. The concept is that of scaling the gradient update by an approximation of the inverse Hessian. Common choices include simple diagonal preconditioners. Although diagonal preconditioners can accelerate convergence in many optimization problems, they cannot provide the fastest convergence rate for imaging problems since they ignore the off-diagonal structure of the Hessian of the objective function (in this context, mainly the response of tomographic systems). Given these premises, strategies that improve the conditioning of the optimization problem have wide applications. Preconditioning is broadly generalizable, and therefore, it can be used in conjunction with other acceleration strategies.

In this article, we propose a new preconditioning strategy and apply it to a gradient descent algorithm with conjugate acceleration. We test it against a subset of the PET Rapid Image reconstruction Challenge (PETRIC) dataset that contains different anthropomorphic phantoms, each of which is representative of different setups encountered in clinical settings (e.g., high/low number of counts per bin, Time-of-flight (TOF) vs non-TOF, cold contrast, and high/low hot contrast).

### 1.2 Non-negatively constrained vs. unconstrained problems

While PET image reconstruction problems are generally discussed as if they are unconstrained, all popular algorithms actually apply a non-negativity constraint to the current estimate $\lambda^{\left(n\right)}$. The original ML-EM algorithm by Vardi et al. (10) uses a multiplicative update, which naturally preserves positivity. The popular BSREM algorithm, which is useful for applying *a priori* regularization strategies, as described by De Pierro and Yamagishi (7), briefly mentions that for elements that “fall below a small threshold, we reset them to the threshold value before proceeding to the next iteration.” This apparently negligible modification modifies the optimization task from an unconstrained to a constrained one, which has different behavior. It is also known that this non-negativity constraint results in bias in the reconstructed image, especially in cold areas [e.g., see Cloquet and Defrise (11)].

Negative numbers in the image could indeed be avoided as PET images represent physical concentration of radioactivity, for which negative values carry no physical meaning. However, they could also be accepted with the understanding that negative numbers are to be interpreted as 0 within the statistical error. The only constraint given by the Poisson log-likelihood is that $\bar{y_{i}}\geq 0\;\forall i$.

## 2 Materials and methods

### 2.1 Reconstruction algorithm

In this article, we decided to allow the solution to be negative. To achieve this, we modified the gradient of the log-likelihood to be

$$\frac{\partial L}{\partial \lambda_{j}}=\sum_{i}a_{i}\;c_{ij}\frac{\bar{y_{i}}-y_{i}}{\max\left(\bar{y_{i}},b_{i}\right)},$$ (3)

The parameter $b_{i}$ in Equation 3 is the additive correction term of the forward model. To see the effect of this modification, we will highlight three cases that show how using $b_{i}$ instead of a generic “small value” allows one to obtain an unbiased solution.

#### 2.1.1 Hot region within the convex hull of the signal

For a pixel j located within the convex hull of the signal (e.g., the body or a phantom), it holds that $\bar{y_{i}}\gg b_{i}\;\forall i\;\;\;\text{s.t.}\;c_{ij}\neq 0$ (i.e., all the LORs that intersect pixel j). In j, both the gradient and the Hessian for a pixel are identical to those of the Poisson log-likelihood. In a “hot” area (i.e., reconstructed activity much higher than 0 compared to the noise), the constraint is not active; therefore, our modified optimization function has identical solution.

#### 2.1.2 Cold region within the convex hull of the signal

Given the considerations highlighted above about $\bar{y_{i}}$ for pixels within the convex hull of an object, in a cold region (i.e., $\lambda_{j}\approx 0$), the gradient and the Hessian of our modified function will be identical to that of the Poisson log-likelihood. Given the absence of a constraint in the image space, $\lambda_{j}$ will be different from that of a constrained problem; however, the expectation of the solution will be that of the ideal Poisson problem, which is constrained in sinogram space (i.e., $\bar{y_{i}}\geq b_{i}$).

#### 2.1.3 Cold region outside the convex hull of the signal

Outside the convex hull of the signal, we have $(\bar{y_{i}}-b_{i})\approx 0$ for all LORs not intersecting it: deviations from the exact Poisson log-likelihood will be found here. Nonetheless, the curvature of the solution remains very close, especially in expectation:

$$\frac{\partial^{2}L}{\partial \lambda_{j}\partial \lambda_{\xi }}=\sum_{i}a_{i}^{2}\;c_{ij}\;c_{i\xi }\frac{y_{i}}{\max{\left(\bar{y_{i}},b_{i}\right)}^{2}},\;E\left(\frac{\partial^{2}L}{\partial \lambda_{j}\partial \lambda_{\xi }}\right)=\sum_{i}a_{i}^{2}\;c_{ij}\;c_{i\xi }\frac{1}{\max\left(\bar{y_{i}},b_{i}\right)},$$

where for all LORs not intersecting the convex hull of the signal $E(\max(\bar{y_{i}},b_{i}))\approx b_{i}$. Given the same expected curvature and a gradient that points to the same minimum (e.g., $\bar{y_{i}}=y_{i}$), we expect the solution, even in this background region, to converge very closely to the values of the exact Poisson likelihood, with the only constraint applied in sinogram space.

It should be noted that if one used $\max(\bar{y_{i}},\alpha b_{i})$, with α<1 or even ≪1, to push $\bar{y_{i}}$ farther away from 0, one would effectively change the expected curvature and bias the solution.

### 2.2 Relative difference prior

It was originally shown by Fessler and Rogers (12) that spatial resolution in regularized emission tomography image reconstruction problems is highly non-uniform. Specifically, hot areas, which in general are those of highest interest, are the most smoothed, given that they have the highest absolute variance in a Poisson experiment (despite the lowest relative variance).

To overcome this, a relative difference prior (RDP) was proposed by Nuyts et al. (13) and has been successfully adopted, even in clinical tomographs. To allow for negative numbers, we defined a slightly modified version as follows:

$$M_{\;j,\xi }=k_{j}k_{\xi }\frac{{\left(\lambda_{i}-\lambda_{\xi }\right)}^{2}}{\sqrt{\lambda_{j}^{2}+\lambda_{\xi }^{2}+\gamma^{2}{\left(\lambda_{j}-\lambda_{\xi }\right)}^{2}\;+\;\varepsilon^{2}}};\;M=\sum_{j}\sum_{\xi \in N_{k}}M_{\;j,\xi },$$ (4)

In Equation 4, $k_{j}$ are weighting factors depending on the position in the image and j and ξ are two spatially connected pixels.

The gradient of this prior for γ=2 is

$$\frac{\partial M_{\;j,\xi }}{\partial \lambda_{j}}=-\;k_{j}k_{\xi }\;\left(\lambda_{j}-\lambda_{\xi }\right)\frac{2\varepsilon^{2}+6\lambda_{\xi }^{2}-7\lambda_{j}\lambda_{\xi }+5\lambda_{j}^{2}}{{\left(\lambda_{j}^{2}+\lambda_{\xi }^{2}+4{\left(\lambda_{j}-\lambda_{\xi }\right)}^{2}\;+\;\varepsilon^{2}\right)}^{\frac{3}{2}}}.$$

The Hessian $H_{rdp}$ of this prior can also be computed explicitly as follows:

$$\begin{array}{cc} \frac{\partial^{2}M_{\;j,\xi }}{\partial \lambda_{j}^{2}} & =\frac{2\varepsilon^{4}\;-\;\varepsilon^{2}\left(5\lambda_{j}^{2}\;-\;14\lambda_{j}\lambda_{\xi }\;+\;\lambda_{\xi }^{2}\right)\;-\;\lambda_{\xi }^{2}\left(7\lambda_{j}^{2}\;-\;22\lambda_{\xi }\lambda_{j}\;+\;7*\lambda_{\xi }^{2}\right)}{{\left(\lambda_{j}^{2}\;+\;\lambda_{\xi }^{2}\;+\;4{\left(\lambda_{j}\;-\;\lambda_{\xi }\right)}^{2}\;+\;\varepsilon^{2}\right)}^{\frac{5}{2}}}\\ \frac{\partial^{2}M_{\;j,\xi }}{\partial \lambda_{j}\partial \lambda_{\xi }} & =-\frac{2\varepsilon^{4}\;-\;2\varepsilon^{2}\left(\lambda_{j}^{2}\;-\;6\lambda_{j}\lambda_{\xi }\;+\;\lambda_{\xi }^{2}\right)\;-\;\lambda_{\xi }\lambda_{j}\left(7\lambda_{j}^{2}\;-\;22\lambda_{\xi }\lambda_{j}\;+\;7*\lambda_{\xi }^{2}\right)}{{\left(\lambda_{j}^{2}\;+\;\lambda_{\xi }^{2}\;+\;4{\left(\lambda_{j}\;-\;\lambda_{\xi }\right)}^{2}\;+\;\varepsilon^{2}\right)}^{\frac{5}{2}}} \end{array}.$$

### 2.3 The proposed preconditioner

In this article, we present a preconditioning method inspired by Fessler and Booth (14). The original theory was based on a quadratic problem similar to $\left[Ax-y{]}^{T}W[Ax-y\right]$, with A denoting the system matrix and W the covariance matrix. In this article, we extend it to Poisson log-likelihood and 3D systems, introducing further approximations. An ideal preconditioner for a quadratic problem is the inverse of the Hessian. If we can invert the Hessian exactly, the problem would be directly solved. However, this is usually not feasible due to the high computational cost or the non-analytic nature of the inversion, and approximations are needed.

This PET reconstruction problem is not quadratic: the local curvature depends on the current estimate $\lambda^{k}$. Nonetheless, the terms $a_{i};c_{i,j}$ are constant and $\bar{y_{i}}$, a forward projection, is largely influenced by the lowest frequencies of the image; for this reason, the Hessian is approximately constant over all the iterations when provided a reasonable starting point (e.g., a few iterations of OSEM and a low-res filtered backprojection, etc.).

#### 2.3.1 Mixed diagonal-circulant preconditioner

In Fessler and Booth (14), they applied the intuition that in general

$$\sum_{i}a_{i}\;c_{ij}\frac{y_{i}}{{\bar{y}}_{i}^{2}}c_{i\xi }\;a_{i}\approx \hat{\eta_{j}}\;\sum_{i}\;c_{ij}\;c_{i\xi }\;\hat{\eta_{\xi }};\;\hat{\eta_{j}}=\sqrt{\frac{\sum_{i}c_{ij}^{2}\;a_{i}^{2}\frac{y_{i}}{{\bar{y}}_{i}^{2}}}{\sum_{i}c_{ij}^{2}}}.$$ (5)

The approximation in Equation 5 is exact along the diagonal; $\sum_{i}c_{ij}\;c_{i\xi }$ instead is, under general reasonable assumptions for a 2D tomograph, the 1/r spatial frequency filter of the Radon transform. This factorization is useful as it is easy to invert, given that it is the product of two diagonal matrices and a circulant matrix, which is therefore diagonalized by the Fourier transform.

#### 2.3.2 Technical implementation and further approximations

In the original article, a complex method was proposed to approximate the inversion of the circulant matrix, considering a regularization prior that had different “effective strengths” in different parts of the image, which is common for quadratic penalties.

To achieve a more practical preconditioner, we needed to address the following issues: (i) how to estimate $\hat{\eta_{j}}$ given the non-quadratic nature of our problem and the curvature change over iterations; (ii) how to identify an effective alternative to approximate the inversion of $c_{ij}\;c_{i\xi }$, also considering the 3D nature of modern PET scanners; and (iii), how to account for the effect of the prior.

Our final preconditioner will still have the structure shown in Equation 6:

$$s=D\;F^{-1}\;T\;F\;Dg,$$ (6)

where s is the search direction, g is the gradient of the complete log-likelihood, D is a diagonal matrix, F is the Fourier transform, and T is a filter in the spatial frequencies space.

##### 2.3.2.1 Diagonal component of the preconditioner

The matrix D will approximate the inverse of the diagonal of the Hessian. Assuming that the geometrical term $c_{ij}$ is largely Boolean, (i.e., 1 when an LOR intersects a pixel and 0 elsewhere), we state that $\sum_{i}a_{i}^{2}\;c_{i,j}^{2}\approx \sum_{i}a_{i}^{2}\;c_{i,j}$. We also replace $y_{i}/{\bar{y}}_{i}^{2}$ with its expectation $1/\bar{y_{i}}$, with the aim of making $g_{j}$ smoother, even in low-count settings; indeed $(\sum_{i}a_{i}^{2}\;c_{i,j})1/\bar{y_{i}}$ is a back-projection, so $\eta_{j}$ is intrinsically a low-frequency image.

With Equation 7 we finally define the diagonal component of the preconditioner as

$$D_{\;j\xi }=\frac{1}{\eta_{j}}\delta_{\;j\xi },\;\eta_{\;j}=\sqrt{\sum_{i}a_{i}^{2}\;c_{ij}\frac{1}{\bar{y_{i}}}+\sum_{\xi \in N_{j}}\frac{\partial^{2}M_{\;j,\xi }}{\partial \lambda_{j}^{2}}}$$ (7)

with δ being the Kronecker delta.

##### 2.3.2.2 Circulant component of the preconditioner

The other component of the proposed preconditioner is the spatial-frequency filter operation. In the original article, a filter was derived that accounted for the local “effective” strength of the prior and of the tomographic likelihood. Here, under the assumption that the prior used aims for approximately uniform resolution, we assume that the factorization in Equation 5 is identical when including the prior diagonal in $\eta_{j}$. Therefore, we only need to approximate the matrix operation $\sum_{\xi }c_{i,j}c_{i,\xi }\;\phi_{\xi }$ on a generic vector ϕ. We assume that, even in a 3D PET system, the effect of this operation is almost entirely an in-plane 2D low-pass filter (i.e., the typical 1/r blurring), with negligible blurring in the axial direction. The final filter, T, that we propose is defined as follows:

- Build a 1D band-limited Ram-Lak filter impulse response.
- Eventually multiply it by a function to account for the TOF kernel $\exp\left(-x^{2}/4\sigma^{2}\right)$, with σ denoting the TOF resolution ]note the factor 4, as used in Conti et al. (15), to approximate the non-analytical ideal TOF kernel].
- Take the resulting impulse response and compute its Fourier transform.
- Multiply it by a Hamming window with cut-off at the Nyquist frequency [i.e., $0.54-0.46\cos\left(2\pi f/\;f_{\max}\right)$].
- Interpolate the filter in 2D.
- Apply the same 2D filter to all 3D slices independently.

#### 2.3.3 Implementation within a preconditioned conjugate gradient descent algorithm

The final algorithms implemented were as follows, using a preconditioned gradient descent algorithm, with step size estimation. The algorithm was initialized this way:

- Initialize a reconstruction using few OSEM iterations, (14 sub-iterations using 2 subsets) and use it as $\lambda^{0}$.
- Compute the diagonal part of the preconditioner D using this initialization and Equation 7.
- Precompute the filter T.
- Compute the sinogram of the current image estimate ${\bar{y}}^{0}$.

Subsequently, in every iteration k, we perform the following steps:

- Compute the gradient $g^{k}$.
- Compute the preconditioned search direction $s^{k}=DF^{-1}TFDg^{k}$ using Equation 6.
- For k>0 Compute the conjugate search direction using the Polak-Ribière method, as follows:
  $$\gamma^{k}=\max\left(\frac{\left\langle s^{k}\;|\;g^{k}-g^{k-1}\right\rangle }{\left\langle s^{k-1}\;|\;g^{k-1}\right\rangle },0\right);\;s^{k}=s^{k}+\gamma^{k}s^{k-1}.$$
- Compute the forward projection $f_{i}^{k}=\sum_{j}a_{i}\;c_{i,j}\;s_{j}^{k}$.
- Compute the step size by approximating the problem as quadratic and using the tomographic Hessian expectation, as follows:
  $$\alpha^{k}=\frac{\left\langle s^{k}\;|\;g^{k}\right\rangle }{\left\langle f^{k}\;|\frac{1}{\max\left(\bar{y},b\right)}|\;f^{k}\rangle +\beta \langle s^{k}\;|\;H_{rdp}\;|\;s^{k}\right\rangle }$$ (8).
- Update the current estimate of the image and of the sinogram as follows:
  $$\lambda^{k+1}=\lambda^{k}+\alpha^{k}\;s^{k};\;{\bar{y}}^{k+1}={\bar{y}}^{k}+\alpha^{k}\;f^{k}.$$

Note that, by keeping negative numbers in the solution, we can complete one iteration using just one forward and one back projection, despite the need to use a forward projection to compute the quadratic step size for Equation 8. Given the fast nature of this algorithm, we do not employ any subset-based acceleration techniques.

### 2.4 Algorithm testing

Our proposed preconditioner was tested by reconstructing a diverse dataset of PET acquisitions from different PET scanners and different phantoms. All the reconstructions were implemented in SIRF (16), a framework that wraps geometrical routines written in STIR (17).

We used data that were made available during the “PETRIC” challenge (see the official website).

#### 2.4.1 The PETRIC dataset

The PETRIC comprised the following seven datasets:

- **mMR-NEMA**: NEMA-IQ phantom acquired on a Siemens mMR hybrid PET/MR scanner (Siemens Healthineers, Erlangen, Germany). The phantom was filled with a ^18^*F* solution and a 10:1 contrast ratio between the spheres and the background. Data were acquired over 10 min. The phantom contains six spheres with the following diameters: 10, 13, 17, 22, 28, and 37 mm.
- **NEMA-LowCounts**: NEMA-IQ phantom acquired on a Siemens mMR, with lower statistics. The same dataset was re-processed to keep only 1/5 of the total statistics.
- **NEMA-Mediso**: NEMA-IQ acquired on a Mediso Anyscan PET/CT system (Mediso Medical Imaging Systems, Budapest, Hungary). A phantom with the same geometry was realized using ^90^Ge, with a lower 4:1 lesion to background contrast ratio. Acquisition was performed for 1 h.
- **mMR-ACR**: ACR phantom acquired on a Siemens mMR scanner. The phantom, filled with ^18^F, contains multiple hot (spheres) and cold (rods) targets, and it was acquired for 200 s.
- **HBP**: Hoffman 3D Brain Phantom acquired on the Positrigo NeuroLF PET scanner (Positrigo AG, Zurich, Switzerland). The phantom was filled with 39 MBq of ^18^F-FDG and was acquired for 20 min. This scanner has small (3.3 mm) crystals; the phantom represents a human brain glucose distribution, with 4:1 contrast between gray and white matter.
- **Thorax**: Thorax Phantom acquired on the Siemens Vision 600 phantom. This anthropomorphic phantom, filled with ^18^F-FDG, includes multiple areas of different attenuation and lesions with irregular shapes to mimic realistic lesions. This scanner has a TOF resolution of 214 ps. It was acquired for 1,200 s.
- **Vision-HBP**: The Hoffman 3D Brain Phantom acquired on the previously described Siemens Vision 600 scanner (Siemens Healthineers, Erlangen, Germany). This was filled with ^18^F-FDG and acquired for 300 s.

#### 2.4.2 Compared reconstructions

For every dataset, we performed the following four reconstruction strategies:

- **PCG**: the complete proposed method as described in Section 2.3.3.
- **PG**: a preconditioned gradient descent method with the same preconditioner but without using the conjugate search direction strategy.
- **DCG**: conjugate gradient descent with only the diagonal preconditioner (e.g., compared to Equation 6, only doing s=DDg).
- **DG**: applying the same preconditioner as before, without the conjugate search direction.
- **BSREM**: the standard BSREM algorithm to be used as a comparison. It was set up using between five and nine subsets, depending on the scanner’s number of views.

The DG algorithm was run for at least 1,100 iterations to generate reference images. The gradient norm of each step was monitored to ensure that numerical convergence was safely reached. To compare the effect of our unconstrained reconstruction, we ran the same algorithm and also applied a negative value truncation after each update.

Images were also reconstructed using the following three β values: a “mid” value, tuned to provide light smoothing; a value three times higher that resulted in a “high” prior strength; and a “low” value of 1/3 the “mid” one, where the influence of the prior is almost negligible. This was done to test the effectiveness of our preconditioner at different levels of relative importance of the prior Hessian vs. the tomographic problem one.

##### 2.4.2.1 Metrics

To analyze the reconstruction speed and compare multiple reconstructions when necessary, we used the following metrics:

- Root mean squared error (RMSE): root mean squared error between the pixels of the two images x and $\lambda_{\mathrm{ref}}$. This value was normalized to the mean activity value within the “whole object mask.” $\mathrm{RMSE}=(\sqrt{\sum_{i}^{N}(x_{i}-\lambda_{\mathrm{ref}\;i}{)}^{2}/N})/\left\langle \lambda_{\mathrm{ref}}\right\rangle$.
- Volume of interest (VOI) error: relative error between the mean activity within a VOI in two images $\text{VOI\textbackslash{}\_E}=(\sum_{i\in \mathrm{VOI}}x_{i}/\sum_{i\in \mathrm{VOI}}\lambda_{\mathrm{ref}\;i})-1.$ In the PETRIC dataset, for each phantom, a different number of pre-established VOIs were provided by the organizers (min: 3; max: 6). Some phantoms (mMR-ACR and HBP) also included cold VOIs.

These metrics were plotted as a function of “epochs,” with one epoch representing a complete pass over the whole dataset, to allow a fair comparison between BSREM and the proposed unaccelerated algorithms.

## 3 Results

### 3.1 Impact of negative numbers on the solution

In Table 1, we report the RMSE between a positively constrained reconstruction and our proposed unconstrained reconstruction. The RMSE was computed within the “whole object mask,” as we are interested in the differences between the images within the convex hull of the activity. In general, values smaller than 2% were observed. The highest value was found in the Thorax and HBP phantoms, which included cold areas. We also report, among all the ROIs, the highest and lowest absolute error for each phantom, which showed very high levels of agreement, even for phantoms that included cold VOIs.

**Table 1:** Comparison of positively constrained reconstructions vs. unconstrained reconstructions showing the standard deviation between the reconstructions and the highest and lowest absolute error values between regions of interest.

Phantom	RMSE (%)	Min VOI AE (%)	Max VOI AE (%)
mMR-NEMA	0.8	0.02	0.14
NEMA-LowCounts	1.0	0.00	0.03
NEMA-Mediso	1.9	0.03	0.43
mMR-ACR	1.0	0.03	0.42
HBP	0.5	0.09	0.39
Thorax	2.0	0.00	0.02
Vision-HBP	3.1	0.07	1.3

### 3.2 Convergence speed comparison

**Figure 1:**
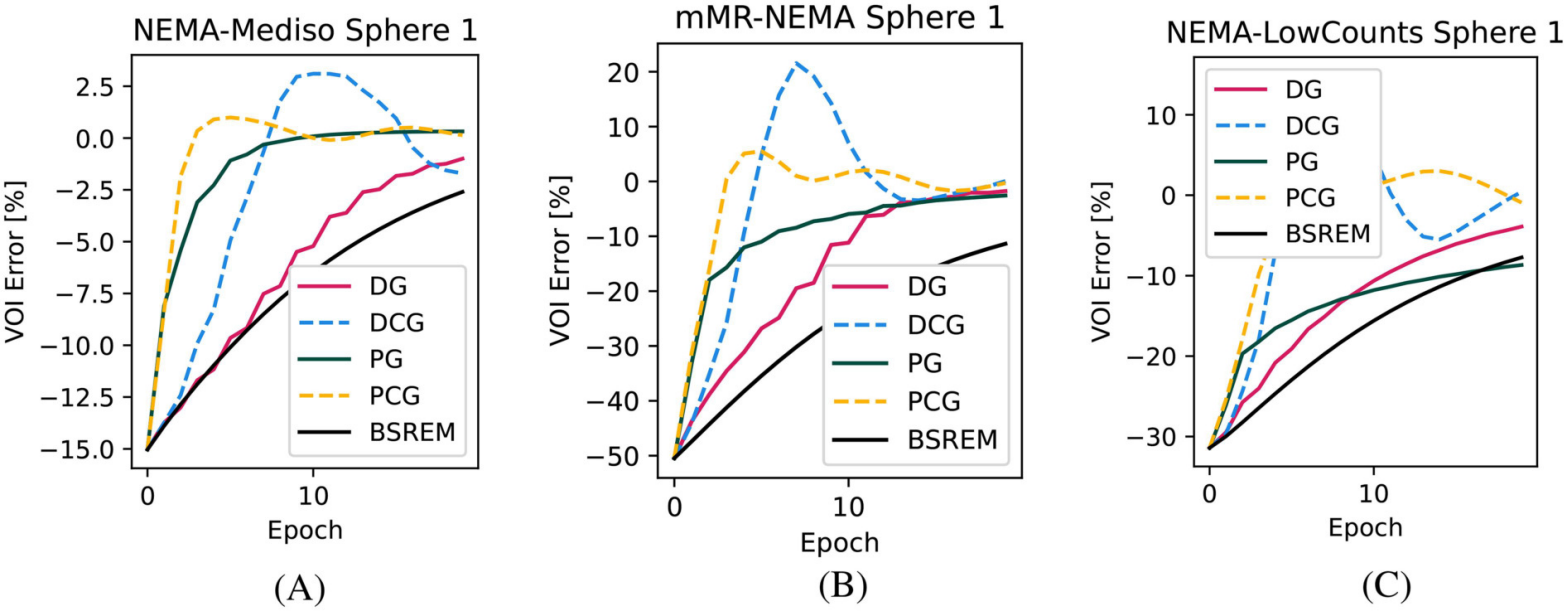
Convergence plots of the smallest (10 mm) sphere for the three NEMA-IQ phantoms from the PETRIC. **(A)** Mediso scanner, 4:1 contrast, **(B)** mMR scanner, 10:1 contrast, and **(C)** same but with 1/5th of the counts.

In Figure 1, we show the convergence speed with the four tested algorithms for the smallest sphere of the NEMA-IQ phantom in the three different acquisitions where it was used (mMR-NEMA, NEMA-LowCounts, and NEMA-Mediso). With the PCG algorithm, a near convergence value was obtained in less than 10 iterations, while with BSREM, 20 iterations were still clearly insufficient.

**Figure 2:**
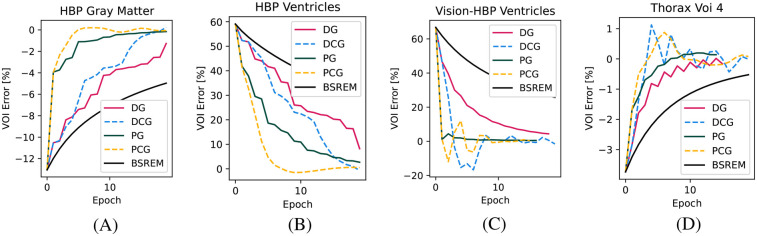
Convergence plots for the other three relevant VOIs. **(A)** HBP gray matter, **(B)** HBP ventricles, **(C)** HBP ventricles acquired on the Vision-600 TOF scanner, and **(D)** Thorax phantom VOI 4.

In Figure 2, we show the convergence for the four other VOIs, including two cold VOIs (ventricles in the HBP phantom), with one characterized by many high-frequency details (gray matter in the HBP phantom) and a realistic lesion in the Thorax phantom. Similarly to above, in these cases, we found the fastest convergence with the PCG algorithm. Especially in the cold regions, the superiority of the proposed algorithm compared to BSREM can clearly be appreciated.

**Figure 3:**
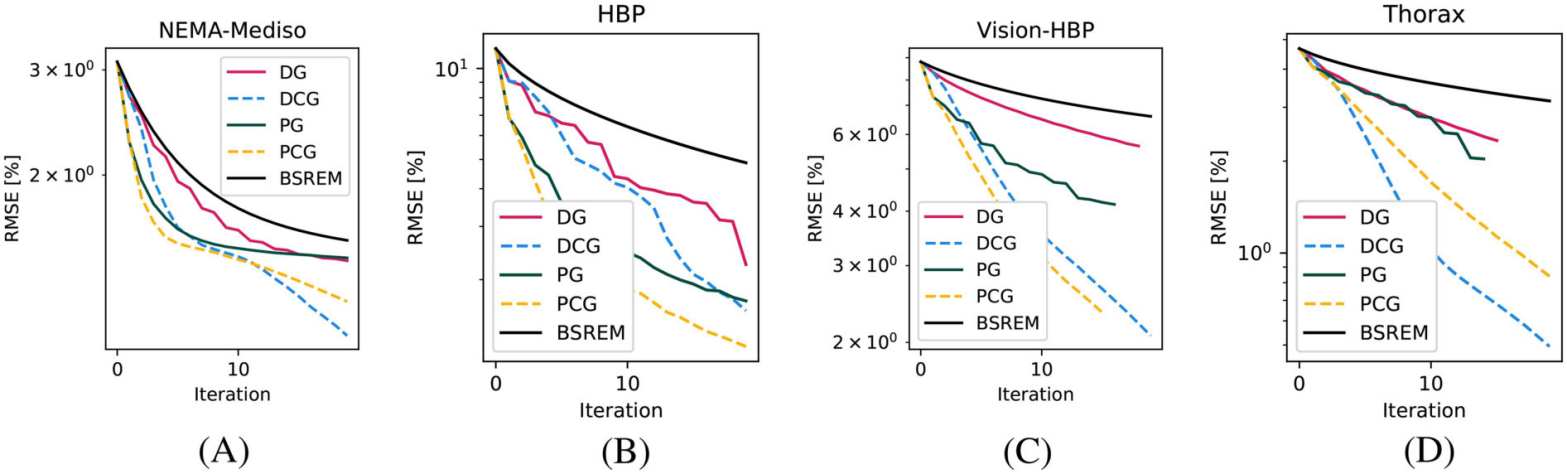
RMSE convergence plots for four phantoms. **(A)** MEDISO IQ, **(B)** HBP, **(C)** HBP acquired on the Vision-600 TOF scanner, and **(D)** Thorax phantom.

In Figure 3, we show the RMSE of the whole image for four acquisitions with different properties, which show the advantage in all contexts of using both the diagonal and circulant preconditioners of varying magnitudes.

### 3.3 Impact of the prior’s strength

**Figure 4:**
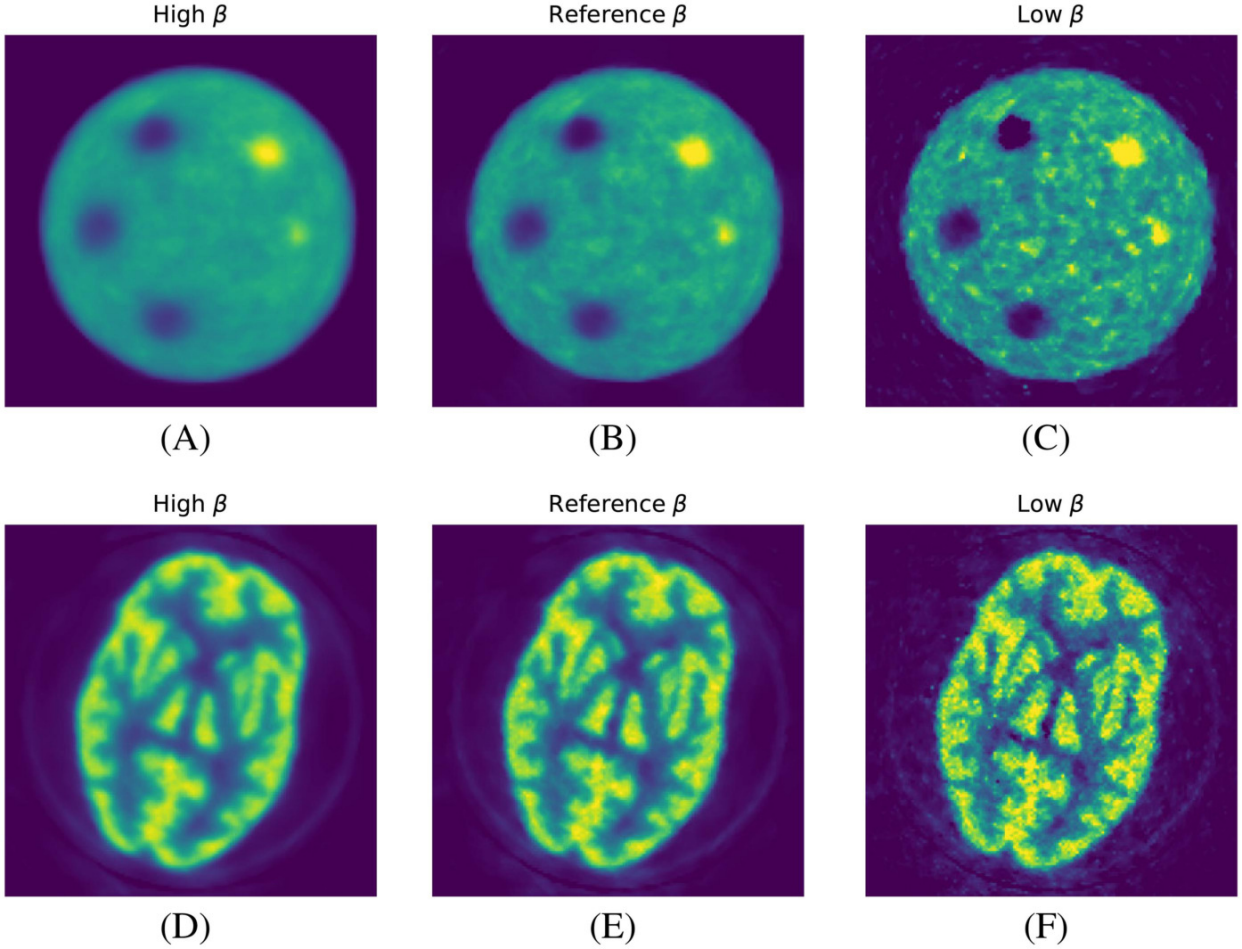
Qualitative images from the reconstruction of two phantoms as a function of β strength. **(A**–**C)** ACR phantom and **(D**–**F)** HBP phantom. Left to right: high, reference, and low β.

**Figure 5:**
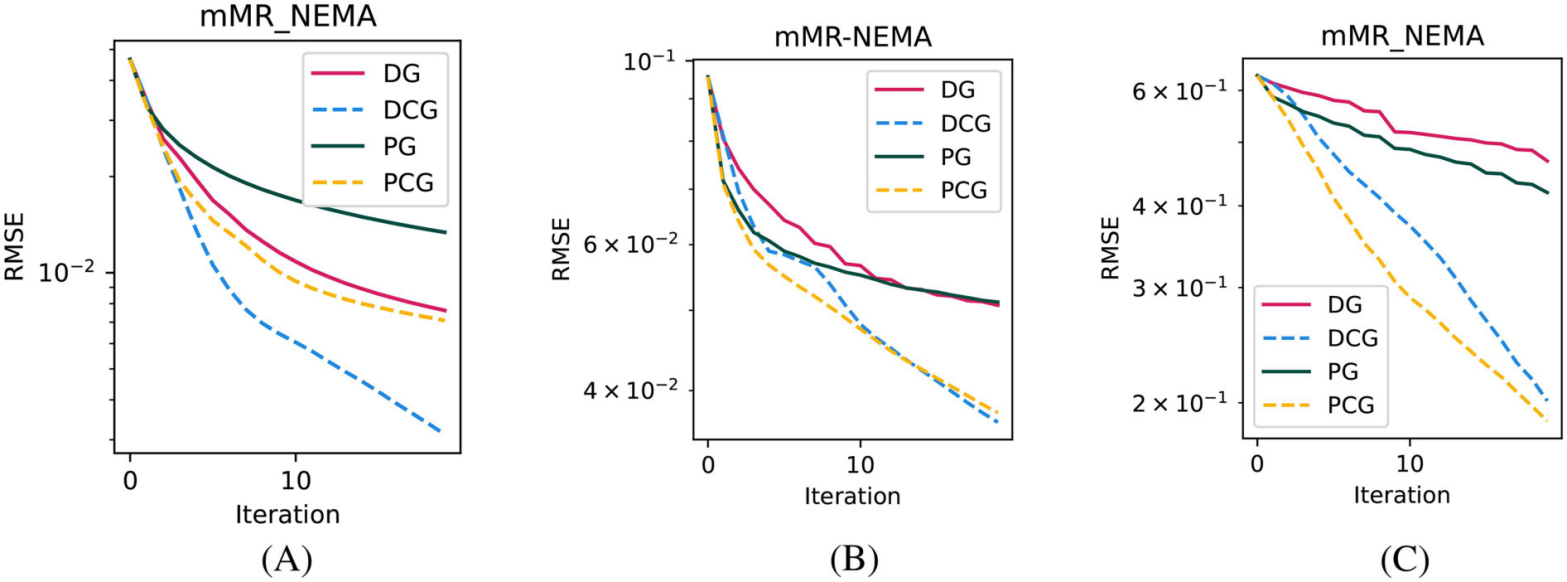
RMSE plot as a function of β for the mMR-NEMA phantom. **(A)** High β, **(B)** reference high β, and **(C)** low β.

**Figure 6:**
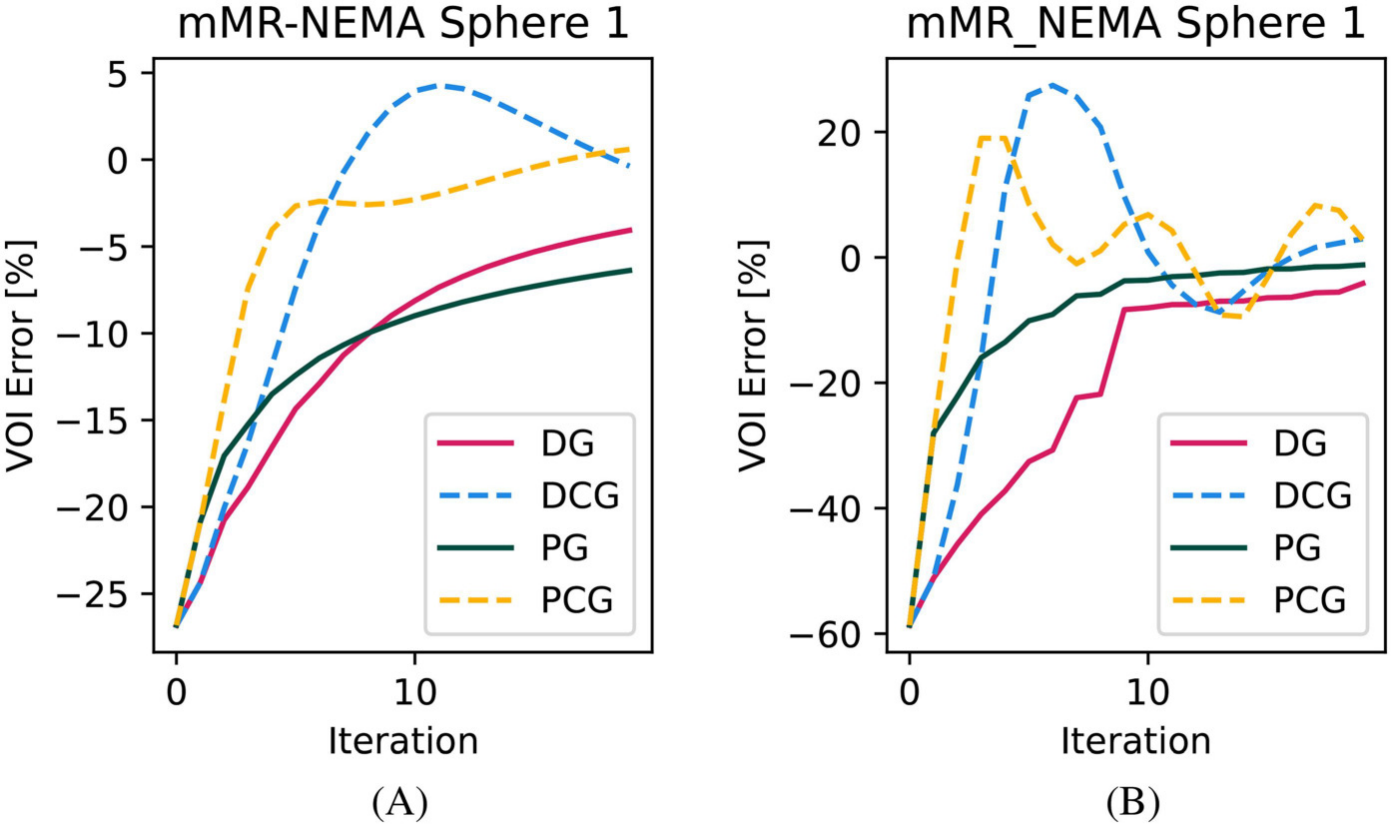
Convergence plot for the NEMA-IQ phantom VOI of the smallest sphere. **(A)** High β and **(B)** low β.

In Figure 4, we show the qualitative images of the reconstructions obtained using the three β values, showing how effective the RDP is in noise suppression. The β value impacts the effectiveness of the circulant part of the preconditioner, especially for the RMSE of the whole object. This is shown for the mMR-NEMA phantom in Figures 5, 6. At the low β, the inclusion of the spatial filtering operation maximally improves the convergence speed, while at the highest value, its impact was reduced.

## 4 Discussion

In this article, we proposed a new preconditioner based on one suggested by Fessler and Booth (14). This preconditioner, combining diagonal and circulant parts and implemented as a filter acting on spatial frequencies, is especially effective in a number of diverse test conditions.

First, it should be appreciated that, in all tested conditions, even the smallest targets reached values extremely close to convergence (e.g., <0.5%) in less than 10 iterations, and 4 iterations were often sufficient to get to <1%. In this case, there is no need for subset-based acceleration, guaranteeing convergence to the unique optimal solution without the need for stochastic acceleration techniques, at least if the prior used has a unique solution. Nonetheless, future work can explore the integration of this preconditioner into stochastic algorithms for even faster convergence. Of particular interest is the fact that, by using gradient descent, this convergence is also similar for cold targets, as can be appreciated in the comparison with BSREM. Such regions are a major problem for multiplicative algorithms [e.g., see Presotto et al. (2)]. In this article, we also used an unconstrained reconstruction approach. Beyond faster optimization of the problem, this should also be welcome as it removes the bias in cold regions, which is known to hinder quantitative PET applications.

An analysis of the behavior of the proposed preconditioner results in some interesting findings. The full preconditioner, including the spatial filter, first improved the convergence of the activity value in the VOIs for all conditions (phantoms and β values). The RMSE of the whole object, which also weighs background regions, often plateaus for a couple of iterations, especially at higher β values. This can be explained by difficulties in following search directions containing frequencies suppressed by the filter. Combining the preconditioner with conjugate search directions improved this. Improving the filter design and changing its cut-off frequency as a function of β could further improve it. It should be remembered that the solution implied by the relative difference prior still does not have uniform spatial resolution: for activity values lower than ε, the solution is effectively unregularized, and the edges between regions with contrast higher than γ are —intentionally— preserved and not smoothed. It was noticeable, indeed, that introducing the filter provided the highest acceleration with lower β values, where the approximation made in neglecting the effect of the prior was less influential.

In the reconstruction of the Thorax dataset, acquired on a TOF scanner with very high temporal resolution, the application of the filter provided only marginal improvements (which indeed can only be seen in the RMSE plots in the first iteration). This can be expected given that the better the TOF resolution, the lower the filter strength.

This article opens the possibility for further research that can be achieved by further optimizing the relative difference prior and making it better behaved, and also by fine-tuning the filtering part of the preconditioner. While it was studied here using a conjugate gradient descent, the same can be applied to any optimization algorithm or other acceleration strategies (e.g., Primal-dual and Nesterov).

The main innovations presented in this article can be summarized as follows:

- We used an approximate expectation of the tomographic Hessian instead of its observed value, which leads to a smooth image.
- We used an easy-to-compute approximated 2D filter as compared to the one proposed by Fessler and Booth (14), which would have required a 3D extension and a less generalizable approach.
- We allowed the solution to contain negative numbers, to allow for faster convergence.

## 5 Conclusions

In this article, we proposed a preconditioning strategy that, when implemented in a conjugate gradient descent algorithm with step size estimation, allows for effective convergence to be reached in less than 10 iterations over the whole dataset, even in the usually challenging cold regions.

## Data Availability

The datasets analyzed for this study can be found in the PETRIC challenge repository at https://github.com/SyneRBI/PETRIC. The code developed has been uploaded in this dedicated GitHub repository: https://github.com/SyneRBI/PETRIC-Tomo-Unimib/tree/Frontiers-paper.

## References

[1] Tong S Alessio AM Kinahan PE. Image reconstruction for PET/CT scanners: past achievements and future challenges. Imaging Med. (2010) 2:529. 10.2217/iim.10.49 21339831 PMC3039307

[2] Presotto L Bettinardi V De Bernardi E. A simple contrast matching rule for OSEM reconstructed PET images with different time of flight resolution. Appl Sci. (2021) 11:7548. 10.3390/app11167548

[3] Qi J Leahy RM. Iterative reconstruction techniques in emission computed tomography. Phys Med Biol. (2006) 51:R541. 10.1088/0031-9155/51/15/R01 16861768

[4] Qi J Leahy RM. Resolution and noise properties of MAP reconstruction for fully 3-D PET. IEEE Trans Med Imaging. (2000) 19:493–506. 10.1109/42.870259 11021692

[5] Lindström E Sundin A Trampal C Lindsjö L Ilan E Danfors T, et al. Evaluation of penalized-likelihood estimation reconstruction on a digital time-of-flight PET/CT scanner for ^18^F-FDG whole-body examinations. J Nucl Med. (2018) 59:1152–8. 10.2967/jnumed.117.200790 29449445

[6] Hudson HM Larkin RS. Accelerated image reconstruction using ordered subsets of projection data. IEEE Trans Med Imaging. (1994) 13:601–9. 10.1109/42.363108 18218538

[7] De Pierro AR Yamagishi MEB. Fast EM-like methods for maximum “a posteriori” estimates in emission tomography. IEEE Trans Med Imaging. (2001) 20:280–8. 10.1109/42.921477 11370895

[8] Defazio A Bach F Lacoste-Julien S. SAGA: a fast incremental gradient method with support for non-strongly convex composite objectives. In: Cortes C Welling M Ghahramani Z Weinberger KQ Lawrence ND, editors. Advances in Neural Information Processing Systems. Montreal, QC: Neural Information Processing Systems Foundation (2014). p. 1646–54.

[9] Twyman R Arridge S Kereta Z Jin B Brusaferri L Ahn S, et al. An investigation of stochastic variance reduction algorithms for relative difference penalized 3D PET image reconstruction. IEEE Trans Med Imaging. (2023) 42:29–41. 10.1109/TMI.2022.3203237 36044488

[10] Vardi Y Shepp LA Kaufman L. A statistical model for positron emission tomography. J Am Stat Assoc. (1985) 80:8–20. 10.1080/01621459.1985.10477119

[11] Cloquet C Defrise M. MLEM and OSEM deviate from the Cramer-Rao bound at low counts. IEEE Trans Nucl Sci. (2012) 60:134–43. 10.1109/TNS.2012.2217988

[12] Fessler JA Rogers WL. Spatial resolution properties of penalized-likelihood image reconstruction: space-invariant tomographs. IEEE Trans Image Process. (1996) 5:1346–58. 10.1109/83.535846 18285223

[13] Nuyts J Bequé D Dupont P Mortelmans L. A concave prior penalizing relative differences for maximum—a-posteriori reconstruction in emission tomography. IEEE Trans Nucl Sci. (2002) 49:56–60. 10.1109/TNS.2002.998681

[14] Fessler JA Booth SD. Conjugate-gradient preconditioning methods for shift-variant pet image reconstruction. IEEE Trans Image Process. (1999) 8:688–99. 10.1109/83.760336 18267484

[15] Conti M Bendriem B Casey M Chen M Kehren F Michel C, et al. First experimental results of time-of-flight reconstruction on an LSO PET scanner. Phys Med Biol. (2005) 50:4507. 10.1088/0031-9155/50/19/006 16177486

[16] Ovtchinnikov E Brown R Kolbitsch C Pasca E da Costa-Luis C Gillman AG, et al. SIRF: synergistic image reconstruction framework. Comput Phys Commun. (2020) 249:107087. 10.1016/j.cpc.2019.107087

[17] Thielemans K Tsoumpas C Mustafovic S Beisel T Aguiar P Dikaios N, et al. STIR: software for tomographic image reconstruction release 2. Phys Med Biol. (2012) 57:867. 10.1088/0031-9155/57/4/867 22290410

